# Functional gut microbiota dynamics of generalist and specialist bacteria in association with chicken growth

**DOI:** 10.1093/ismeco/ycag091

**Published:** 2026-04-10

**Authors:** Sofia Marcos, Iñaki Odriozola, Ostaizka Aizpurua, Raphael Eisenhofer, Sarah Siu Tze Mak, Garazi Martin-Bideguren, Varsha Kale, Germana Baldi, Lorna J Richardson, Robert D Finn, Joan Tarradas, Andone Estonba, M Thomas P Gilbert, Antton Alberdi

**Affiliations:** Applied Genomics and Bioinformatics, University of the Basque Country (EHU), Sarriena z/g, 48940 Leioa, Spain; Center for Evolutionary Hologenomics, Globe Institute, University of Copenhagen (UCPH), Øster Voldgade 5-7, 1350 Copenhagen, Denmark; Center for Evolutionary Hologenomics, Globe Institute, University of Copenhagen (UCPH), Øster Voldgade 5-7, 1350 Copenhagen, Denmark; Center for Evolutionary Hologenomics, Globe Institute, University of Copenhagen (UCPH), Øster Voldgade 5-7, 1350 Copenhagen, Denmark; Center for Evolutionary Hologenomics, Globe Institute, University of Copenhagen (UCPH), Øster Voldgade 5-7, 1350 Copenhagen, Denmark; Center for Evolutionary Hologenomics, Globe Institute, University of Copenhagen (UCPH), Øster Voldgade 5-7, 1350 Copenhagen, Denmark; Center for Evolutionary Hologenomics, Globe Institute, University of Copenhagen (UCPH), Øster Voldgade 5-7, 1350 Copenhagen, Denmark; European Molecular Biology Laboratory, European Bioinformatics Institute (EMBL-EBI), Wellcome Genome Campus, Hinxton, Saffron Walden CB10 1SD, United Kingdom; European Molecular Biology Laboratory, European Bioinformatics Institute (EMBL-EBI), Wellcome Genome Campus, Hinxton, Saffron Walden CB10 1SD, United Kingdom; European Molecular Biology Laboratory, European Bioinformatics Institute (EMBL-EBI), Wellcome Genome Campus, Hinxton, Saffron Walden CB10 1SD, United Kingdom; European Molecular Biology Laboratory, European Bioinformatics Institute (EMBL-EBI), Wellcome Genome Campus, Hinxton, Saffron Walden CB10 1SD, United Kingdom; Animal Nutrition, Institute of Agrifood Research and Technology (IRTA), 43120 Constantí, Catalonia, Spain; Applied Genomics and Bioinformatics, University of the Basque Country (EHU), Sarriena z/g, 48940 Leioa, Spain; Center for Evolutionary Hologenomics, Globe Institute, University of Copenhagen (UCPH), Øster Voldgade 5-7, 1350 Copenhagen, Denmark; University Museum NTNU, Erling Skakkes gate 47B, 7012 Trondheim, Norway; Center for Evolutionary Hologenomics, Globe Institute, University of Copenhagen (UCPH), Øster Voldgade 5-7, 1350 Copenhagen, Denmark

**Keywords:** broiler chickens, caecal microbiota, multi-omics, functional microbiota dynamics

## Abstract

The early-life development of the gut microbiome in broiler chickens is a dynamic ecological process with significant implications for host physiology and productivity. Using 388 genome-resolved metagenomic and 61 metatranscriptomic samples across two replicated trials, we analysed the compositional and functional succession of the caecal microbiome in chickens from hatching to slaughter age. We reconstructed 822 bacterial genomes and distilled gene annotations into comprehensive metabolic traits that captured the functional capacities of each genome. We observed that the increase in microbial diversity with chicken age was accompanied by a decline in community-level average metabolic capacity, driven by a shift from metabolically versatile generalists (Lachnospiraceae) to hitherto uncultured, genome-reduced specialists (RF39, RF32, and UBA1242). However, the specific identity of the dominant genome-reduced specialists varied among individuals, resulting in contrasting associations with host body weight. At slaughter age, only 10 UBA660 (RF39) bacteria were positively associated with body weight, while other genome-reduced lineages, such as UBA1242 (Christensenellales), were among 190 negatively associated bacteria. Gene expression analyses revealed that despite their reduced functional repertoire, UBA660 exhibited greater metabolic activity than UBA1242, particularly in the production of two key metabolites for host nutrition and intestinal homeostasis: the essential amino acid lysine and the signaling molecule indole-3-acetate. These findings provide new insights into the functional ecology of the chicken gut microbiome and highlight the relevance of cultivation approaches to retrieve underexplored and uncultured bacterial taxa, which could open new avenues for microbiome-based strategies aimed at improving poultry growth and health in intensive production systems.

## Introduction

Microbial primary succession coincides with significant developmental changes in the host, fostering complex bidirectional feedback loops between the host and its microbiome [1, 2]. Correct immune-microbiota interactions during host development are essential to ensure optimal adult stages, but also to guarantee short-term health [3, 4]. The latter is especially evident in animals raised in intensive production systems [5, 6].

Broiler chickens bred for meat experience a sixty-fold body mass increase in the five weeks between hatching and slaughtering [7], a period of time in which birds do not reach sexual maturity [8] and early microbial interactions affect host physiological changes [9]. Likewise, their gut microbial communities develop without maternal-offspring transmission, making their maturation highly unstable and susceptible to pathogens [8]. Under this highly variable scenario, antibiotics have been historically used as growth promoters and to keep pathogens in check [10]; but since increasing restrictions, microbe-based products have emerged as an alternative strategy to achieve more efficient and sustainable farming practices [11]. However, the task of designing effective microbe-based solutions is challenged by our limited knowledge on the functional underpinnings of how chicken microbiomes develop [12].

Ecological and physiological characterization of bacteria has traditionally relied on cultivation and targeted sequencing-based approaches [13, 14]. Amplicon-based studies have revealed a characteristic pattern of taxonomic changes that take place during the first weeks of a chicken's life [15–18]. Studies have associated microbial diversity [19] and specific taxa [20] with chicken growth performance; although only very few findings have been reproduced in independent trials [21]. Nevertheless, these methods have only explored a small fraction of the immense bacterial diversity [13] and are limited in their ability to make direct functional inferences [14], hindering our potential to uncover the underlying biological processes of early life microbiome assembly [22, 23].

The direct functional insights offered by genome-resolved metagenomics represent a significant qualitative advancement in the analysis of microbial community functioning [24–28]. Characterizing the functional traits of microbial communities enhances our ability to apply ecological theory to microbiome dynamics, enabling, for instance, the investigation of the dynamics of metabolic generalists and specialists across microbiome development and in response to environmental variables. Metabolic generalists carry broad enzyme repertoires that usually result in higher metabolic independence from other bacteria. Specialists, in contrast, are limited to a narrower set of metabolic functions, relying on a limited range of substrates, and therefore often adapted to metabolic niches requiring the contribution of other bacteria [22, 23, 29]. If microbial gene expression data is also considered, functional analyses not only reveal what each bacterium and the entire community *could* do, but also what they *actually* do [29].

In light of this, we studied functional dynamics of the caecal microbiome of broilers reared in two replicate trials [12], combining genome-resolved metagenomics and metatranscriptomics with community ecology modeling. We addressed both the relationship between taxonomic characteristics and functional features of microbial communities, as well as their link with chicken growth performance, and discussed implications for microbiome composition dynamics, microbial evolution and animal production.

## Materials and methods

### Animal experiments

Broilers from two genetic lines (Cobb 500 and Ross 308) and both sexes were grown for 35–37 days, simulating intensive farming conditions. Each trial comprised 12 experimental groups (3 treatments x 2 genetic lines x 2 sexes) and two replicates, for a total of 24 pens, each containing 40 animals. Details about the experimental design, procedures and performance results are provided in Tous *et al*. 2022 [12]. A total of six chickens from each pen were euthanized, weighed, and sampled at Days 7–8, 21–22 and 35–37 (multiple days were necessary due to workload, and these differences have been accounted for in the statistical analyses), hereafter simplified to three time points (Days 7, 21 and 35). One of the caecum pouches was isolated and longitudinally opened. The content was gently flushed out and ca. 100 mg of digesta collected for metagenomic and metatranscriptomic analyses. Samples were preserved in DNA/RNA Shield buffer (Zymo Research, USA) and stored at −20°C.

### Data generation

In total, 388 metagenomic and 61 metatranscriptomic samples were sequenced and analysed. DNA and RNA extractions were conducted using a custom purification method [30] optimized for samples preserved on DNA/RNA Shield buffer. Extracted nucleic acids were fragmented to an average length of 400 nucleotides using a Covaris LE220 ultrasonication device. The standard amount of DNA imputed to the BEST [31] ligation-based library preparation was 200 ng, using BGI adapters [32]. The success of the library preparation process was assessed by qPCR assays, through which the optimal number of cycles to achieve the desired DNA molarity while reducing clonality was estimated (see Supplementary Methods). Sequencing was performed at BGI (Shenzen, China) in multiple MGISeq-2000 runs with 150 bp paired-end chemistry. Sequencing effort per sample typically varied between 8GB and 16GB (26 and 52 million reads).

For microbial metatranscriptomic analyses, rRNA depletion was performed using TIANSeq rRNA Depletion Kit (Cat.No. NR101-T1), after which cDNA conversion was carried out with random hexamers and Illumina short-read sequencing libraries were prepared using Novogene NGS RNA Library Prep Set (PT042). Library molarities were checked with Qubit and real-time PCR for quantification and bioanalyzer for size distribution detection. Quantified libraries were pooled and sequenced on an Illumina NovaSeq 6000 platform with 150 bp paired-end chemistry, aiming for 5GB of protein-coding gene data.

### Bioinformatic data processing

#### Functional annotation and distillation of MAG catalogue

Using a subset of 261 deeply sequenced samples, we generated a catalogue encompassing 822 MAGs (a summary overview can be found in Supplementary Methods, with the MAG assembly statistics in Supplementary Table S1). Taxonomy annotation and phylogenetic tree preparation of the MAG catalogue were performed using GTDB-Tk [33]. The MAGs were functionally annotated by the ensemble approach implemented in DRAM [34], which includes Pfam [35], KEGG [36], UniProt [37], CAZY [38], and MEROPS [39] databases. We then used the R package distillR (https://github.com/anttonalberdi/distillR) to transform raw annotations into quantitative genome-inferred functional traits (GIFTs). DistillR contains a set of >300 metabolic curated pathways and modules derived from KEGG [36] and MetaCyc [40] databases, which are used to obtain quantitative estimates of the metabolic capacities of microorganisms through quantifying the relative representation of genes required for accomplishing a metabolic task. GIFTs range between 0 and 1, the zero indicating none of the genes defined in the pathway are present in the genome and one indicating that all genes are present. When a step within a pathway requires the presence of two identifiers, the step is considered full if both identifiers are present, half full if one is present and empty if none is present. We measured 170 GIFTs per genome (complete detailed list can be found in Supplementary Table S3), whose values were averaged to obtain a genome-level mean metabolic capacity metric, hereafter referred to as Metabolic Capacity Index (MCI). Based on the MCI values, MAGs were classified as low (<0.15), medium (0.15–0.30), or high (>0.30) capacity bacteria.

#### Metagenomic data processing and read mapping

Sequencing adapters and exact duplicates were removed using AdapterRemoval 2.2.4 [41] and seqkit 0.7.1 [42]. Sequences were mapped to the latest chicken reference genome (galGal6, NCBI Assembly accession GCF_000002315.6) using bwa [43], increasing the minimum seed length to 25 in order to reduce the number of incorrectly aligned read pairs from the metagenomic fraction. Aligned reads were quality assessed, sorted and the metagenomic fraction was filtered out using SAMtools 1.11 [43]. Last, metagenomic reads were mapped to the MAG catalogue using bwa and further summarized with SAMtools. The mapping success of the caecum metagenomic reads against the bacterial catalogue was 67 ± 6% (Supplementary Table S2), indicating that we obtained a nearly complete representation of the caecal bacterial community. Read-mapping counts resulting in <30% genome coverage per sample were removed from further analysis. Retained read-mapping counts were divided by the total number of paired-reads per sample, and multiplied by 100 to give the percentage of reads mapped to the MAG catalogue for each sample. Relative abundance was estimated by adapting the RPKG (Reads Per Kilobase per Genome equivalent) formula [44]. It is referred to as RPMM (Reads Per Million bases of genome, per Million mapped reads), as reads mapped to MAGs were normalized both by genome length (divided by 1 M) and by read length (divided by 1 M).

### Metatranscriptomic data processing

A custom snakemake [45] pipeline was used for the metatranscriptomic data processing (https://github.com/anttonalberdi/holoflow/tree/EisenRa/workflows/metatranscriptomics). Reads were trimmed and quality controlled using fastp [46], keeping reads >60 bp and with Phred scores >20. Processed reads were then mapped against the host genome (galGal6) using STAR [47]. The unmapped reads were then mapped to a combined database containing the SILVA 16S rRNA SSU and LSU NR 99 [48], and the 5SRNAdb [49] using Bowtie2 [49] with default parameters. Unmapped reads were then mapped to the MAG catalogue genes (outputted from DRAM; genes.fna.gz) using Bowtie2 with default parameters. Finally, the gene read counts were calculated using CoverM [50], requiring both pairs of reads to hit the gene (proper-pairs-only flag).

### Data analysis

Metagenomic counts were standardized by MAG length. Alpha diversity measurements were calculated using neutral, phylogenetic and functional Hill numbers at the order of magnitude q = 1, thus weighing MAGs according to their relative abundances, using the R package hillR [51]. Beta diversity (dissimilarity) metrics were generated for the same order of magnitude using the same package. The phylogenetic tree employed to compute phylogenetic metrics was derived from the GTDB [52] tree constructed by GTDB-tk [33] for taxonomic annotation, after pruning tips of reference genomes using the R package ape [53]. The functional diversity analyses were based on a MAG trait matrix including pathway fullness values of 350 KEGG modules generated with DRAM [34].

To explore the landscape of the functional capabilities of the chicken caecum microbiome, the bacterial MAGs were ordinated based on their GIFTs through a PCoA analysis using Euclidean distances calculated with the R package vegan [54].

To assess the temporal development of different components of microbial alpha diversity metrics, we used linear mixed effect models (LMM) and analyses of variance (ANOVA) through the R package nlme [55]. Analyses of variance of beta diversities were carried out using permutational analysis of variance (PERMANOVA) through the R package vegan. These tests yielded very weak effects of trial, sex, genetic line and treatment, and none of these categorical variables showed significant interactions with chicken age. Therefore, while considered as covariates in the rest of the analyses, their effect was not interpreted (details in Supplementary Methods).

To visualize the composition of microbial communities across time, distance-based redundancy analysis (db-RDA) was performed using the R package vegan [54].

To identify the MAGs that increased and decreased in abundance with chicken age, MAG counts were analysed using the hierarchical modeling of species communities (HMSC) framework [56], which belongs to the class of joint species distribution models (JSDM) and builds a multivariate generalized linear mixed effect model (GLMM) using Bayesian inference (see Supplementary Methods).

Community-weighted MCI was calculated as the abundance-weighted MCI values of each MAG within each community with distillR, obtained by multiplying each species’ MCI per GIFT by its relative abundance and summing across all species. A mean community MCI was then obtained by averaging community-weighted GIFT values within each individual.

To associate microbiota attributes with animal body weight on Days 21 and 35, we used LMMs. We tested alpha diversity metrics, community-weighted metabolic capacity and community-weighted MAG genome length as response variables. Then, to assess MAG-specific associations with chicken body weight, we fitted a second HMSC model based on the neutral diversity association with chicken body weight that was statistically significant. The model structure was similar to the first HMSC, but in this case, we used data from Day 35 and included trial, chicken age and body weight as fixed effects. Model fitting, evaluation of convergence, assessment of the phylogenetic signal and the evaluation of the significance of the association between MAGs and chicken body weight were also performed similarly to the first HMSC (see Supplementary Methods).

To compare the capabilities to biosynthesise and degrade specific biomolecular compounds between the bacteria positively and negatively associated with chicken body weight, we merged the GIFTs into functional categories (see Supplementary Table S3 for further details) and computed bootstrap 95% confidence intervals around the group averages using the R package boot [57]. Non-overlapping confidence intervals between the species’ groups that positively, negatively and non-significantly associated with chicken body weight were considered as evidence of a significant difference between their functional capabilities. Then, we compared the actual expression of these functions related to the production and degradation of biomolecular compounds between the species positively and negatively associated with chicken body weight. For that, quantitative GIFTs were calculated for both groups of species using the R package distillR, and the expressions of the GIFTs were visualized using a heatmap.

Finally, bacteria with reduced genomes for the last analyses were identified based on a lack of transcription of B0101, B0102, and B0103 functions, which are pathways related to nucleic acid biosynthesis. To compare the genome sizes and average MCI values between the genome-reduced bacteria that were positively and negatively associated with chicken body weight, we applied the same 95% bootstrap confidence interval approach explained above. The expression profiles between both groups were compared by means of a heatmap and by a principal coordinate analysis (PCoA) on a Hellinger-transformed GIFT expression matrix, using the Bray–Curtis dissimilarity index.

## Results

### The complex functional landscape of the chicken caecal microbiome is phylogenetically structured

**Figure 1:**
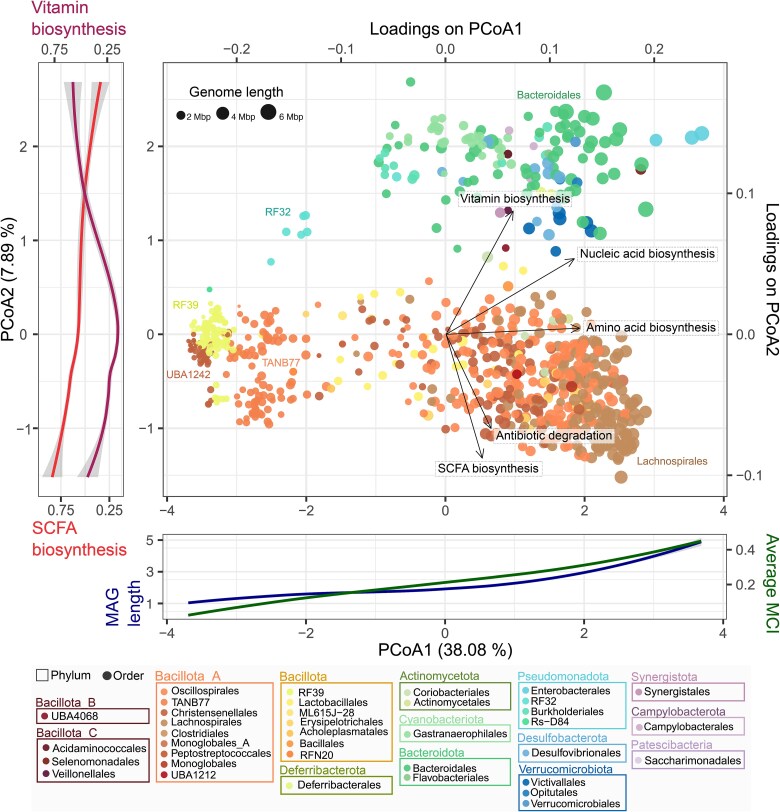
PCoA ordination of bacterial genomes based on their genome-inferred functional traits (GIFTs), colored by taxonomic order. The size of the dots indicates genome length (Mbp). The 2 vectors representing functions that contribute most to the second axis of the ordination are highlighted. The additional two graphics indicate genome length and average metabolic capacity index (MCI) related to the first axis of the PCoA, and vitamin and short chain fatty acids biosynthesis capacity related to the second axis of the PCoA.

Genome ordination based on their GIFTs showed that the functional landscape of the chicken microbiome was phylogenetically structured (Fig. 1), with clusters most clearly separated at order level in the first axis (due to genome length and average metabolic capacity), and at phylum level in the second axis (due to specific differences in functional capacities) of the PCoA. At order level, genome-reduced bacteria with the smallest metabolic capacities appeared close to each other at the left side of the ordination (e.g. RF32, RF39, TANB 77, and UBA1242—members of Christensenellales). Whereas bacteria with big genomes and with the highest metabolic capacities lay on the right side of the ordination (e.g. Lachnospirales, Bacteroidales). At phylum level, Bacillota_A and Bacillota appeared separated from the rest of the clades due to a higher capacity to produce SCFAs and a reduced capacity to biosynthesize vitamins. This distribution reflects a great functional diversity among genomes within the same phylum (Supplementary Fig. S1).

### Microbiome diversity increases while functional capacity decreases with chicken age

**Figure 2:**
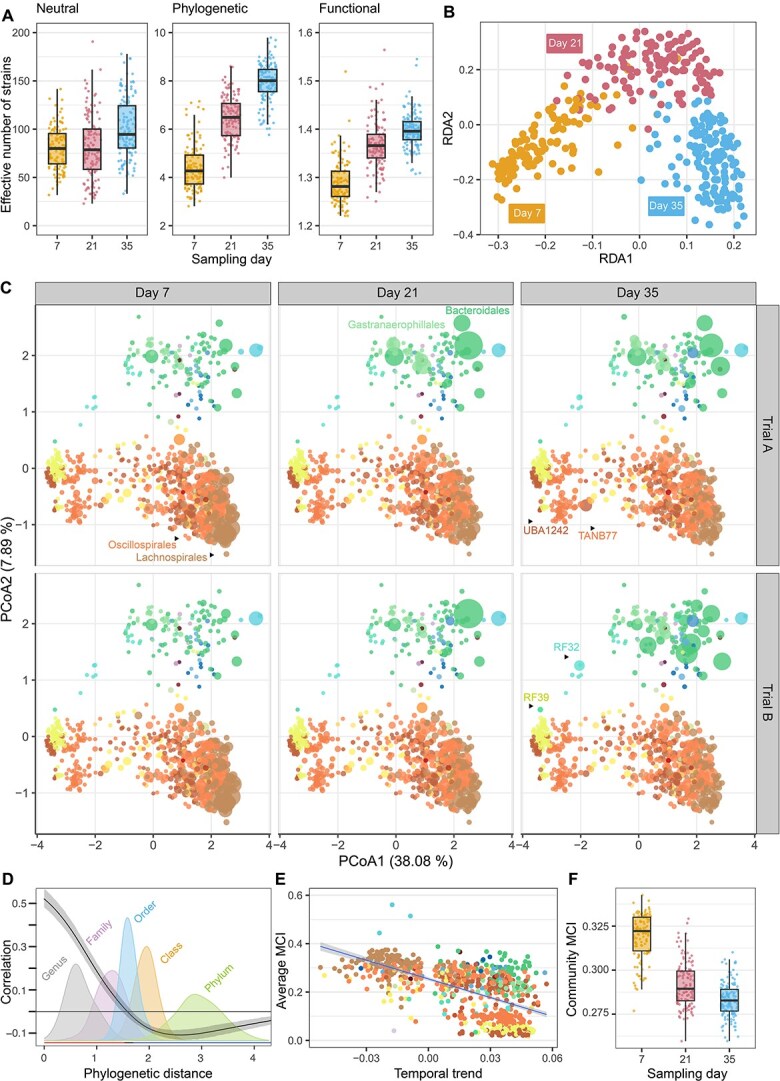
(A) Neutral, phylogenetic and functional diversity metrics across time. ANOVA tables of linear mixed models for different components of alpha diversity are shown in Supplementary Table S4. (B) Distance-based redundancy analysis (dbRDA) plot showing the temporal dynamics of the compositional microbiota variation. PERMANOVA tables for different components of beta diversity are presented in Supplementary Table S5. (C) PCoA ordination of bacterial genomes based on their genome-inferred functional traits (GIFTs), colored by taxonomic order for Days 7, 21 and 35, and trials A and B. The size of the dots represents the relative abundance of each MAG. (D) Phylogenetic correlogram showing the correlation of temporal dynamics across different phylogenetic distances, expressed over density curves of the main taxonomic levels. (E) Correlation between average metabolic capacity index (MCI) of each MAG and its response to time according to HMSC results. (F) Average community-level MCI values for each individual across time.

The analysis of the temporal dynamics of the microbiome of 388 broilers at 7, 21, and 35 days across two replicate trials revealed profound changes in community composition, diversity and functionality as chickens grew. All three alpha diversity metrics (neutral, phylogenetic and functional) increased with chicken age (Fig. 2A). Beta diversity analyses also revealed a strong temporal trend, with animals from different trials and experimental groups exhibiting similar trajectories (Fig. 2B, Supplementary Tables S4, S5, S6). Most of the microbiota variation occurred between Days 7 and 21, followed by a more modest change in the following fortnight (Supplementary Fig. S2). At phylum level, the dominance of Bacillota_A declined while the abundance of Bacteroidota increased over time. However, at order level within Bacillota_A, relative abundance of Lachnospirales decreased dramatically, Oscillospirales remained relatively constant, whereas TANB77 and UBA1242 increased over time (Fig. 2C, Supplementary Fig. S3). Lineages including RF39 (phylum Bacillota, class Bacilli), Gastranaerophillales (Cyanobacteriota, Vampirovibrionia), Bacteroidales (Bacteroidota, Bacteroidia), and RF32 (Pseudomonadota, Alphapseudomonadota) also increased steadily over time and shifted from being rare at Day 7 to becoming relatively dominant by Day 35 (Fig. 2C).

Temporal trajectories of each bacterium were modeled using HMSC. In agreement with increased alpha diversities, most bacterial species showed an increasing trend with chicken age: based on a threshold of 0.9 posterior statistical support, 68% of bacterial taxa increased significantly over time, whereas 25% of the taxa decreased. Bacterial dynamics during this entire period showed a very strong phylogenetic signal (ρ = 0.94 with 90% CI [0.93, 0.94]), with bacteria within taxonomic families exhibiting highly similar temporal dynamics compared to more distantly related ones (Fig. 2D). Moreover, the bacteria that lost and gained representation between Days 7 and 35 were functionally structured. There was a clear negative association between the mean metabolic capacity of bacteria and their responses to chicken age (as estimated in bacterium-specific models in HMSC) (Fig. 2E). As a consequence, and despite the observed increase in diversity, the mean community-weighted metabolic capacities of the microbiota decreased over time (Fig. 2F). This was primarily driven by the reduction of most of the taxa with the highest metabolic capacities, such as Lachnospiraceae, and the increase of taxa with mid (e.g. TANB77, Cyanobacteriota, Bacteroidota) to low metabolic capacities (e.g. UBA1242, RF39, RF32).

### Genome-reduced RF39 bacteria positively associate with chicken body weight

**Figure 3:**
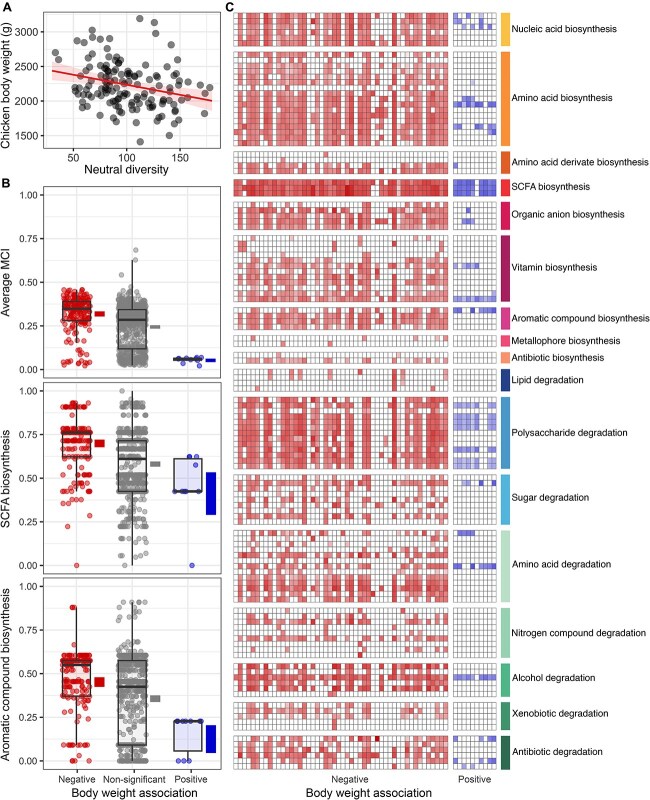
(A) Neutral diversity associated with chicken body weight at Day 35. (B) Average metabolic capacity, and capacity for biosynthesis of SCFAs, and biosynthesis of aromatic compounds, depending on the chicken body weight association classification. Blocks around the group averages indicate bootstrap 95% confidence intervals. (C) Expression profiles of the top 50 negatively and 10 positively associated bacteria with chicken body weight. The 50 negatively associated bacteria were selected from the negative group that contained 190 bacteria based on the magnitude of HMSC’s beta parameter.

To assess any potential relevance of the observed patterns in chicken production, we correlated different microbiota attributes with animal body weight at the slaughter age of 21 and 35 days. These analyses revealed a negative association between animal body weight and neutral alpha diversity (LMM, slope = −2.90, *t*-value = −3.42, *P-*value <.001) (Fig. 3A). The trend was similar across both trials (neutral_div x trial, F-value = 0.006, *P*-value = .93). The association was not significant for phylogenetic and functional alpha diversities. There were also no significant correlations between community-weighted MCI and community-weighted genome length, and chicken body weight at Day 35.

Based on the mentioned association at Day 35, we further explored the associations between specific bacteria and chicken body weight through a second HMSC analysis. As in the case of the temporal trends, the association of MAGs with chicken body weight exhibited a strong phylogenetic signal (ρ = 0.97 with 90% CI [0.96, 0.97]). In agreement with the negative association with neutral alpha diversity, the majority of bacteria exhibited a negative association with chicken body weight: 190 bacteria associated negatively whereas just 10 associated positively. The negatively associated bacteria included genomes with diverse metabolic capacities. Although most of them belonged to Lachnospirales (43%) and Oscillospirales (18%) from phylum Bacillota_A, characterized by high metabolic capacities, the group also contained 18 bacteria with reduced genomes, most of them (11) belonging to the UBA1242 family, Christensenellales order. In contrast, all 10 bacteria that were positively associated with body weight were uncultured RF39 bacteria (family UBA660, genus CAG-460 and CAG-776), which belong to the clade of genome-reduced Bacilli.

Aiming to cast light into the possible mechanistic links between microbiome features and chicken performance, we compared the capabilities to biosynthesize and degrade specific biomolecular compounds between the bacteria that were positively and negatively associated with chicken body weight. The MAGs positively associated with chicken body weight exhibited significantly lower average capacities to produce and degrade most compounds compared to the rest of taxa (Fig. 3B). The only two functions that, despite exhibiting significantly lower values, did not display as pronounced differences as for the other functions were the production of SCFAs (acetate, butyrate and propionate) and, to a lesser extent, the production of indole acetate and aromatic compounds (salicylate, gallate, chorismate, dipicolinate) (Fig. 3B, Supplementary Figs. S4, S5, and Supplementary Table S3).

Analysing the metatranscriptomic data, we compared the actual expression of metabolic functions to produce and degrade biomolecular compounds between the groups that were positively and negatively associated with body weight. The results confirmed that whereas the negatively associated group was dominated by bacteria exhibiting activity in a wide range of functional capabilities, the 10 positively associated UBA660 bacteria had very low or no activity for most of the biosynthetic and degradative pathways (Fig. 3C). However, the results confirmed that positively associated bacteria exhibited elevated expression of SCFA production pathways, while revealing significant activity in several polysaccharide degradation pathways (Fig. 3C).

**Figure 4:**
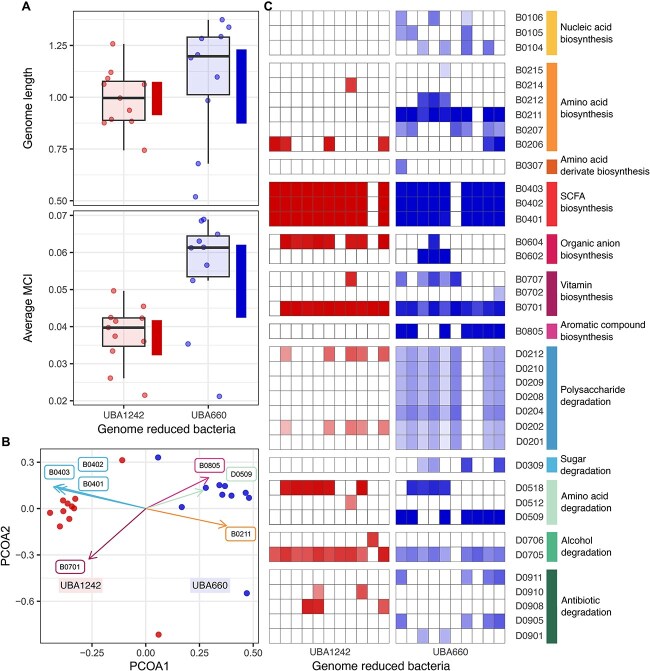
(A) MAG length (Mbp) and average metabolic capacity index (MCI) comparison between genome-reduced MAGs (UBA660 and UBA1242) that were positively and negatively associated with chicken body weight. Blocks around the group averages indicate bootstrap 95% confidence intervals. (B) PCoA ordination of gene expression profiles of UBA660 and UBA1242. The vectors representing functions that contribute most to the ordination are highlighted: SCFA (B04), vitamin B1 (B0701), amino acid lysine (B0211) and indole-3-acetate (B0805) biosynthesis; and arginine degradation (D0509). (C) Gene expression profiles of UBA660 and UBA1242 aggregated by GIFTs. The meaning of the codes can be found in Supplementary Table S3.

We further explored the genomic and functional characteristics of genome-reduced bacteria that were positively (UBA660) and negatively (UBA1242) associated with chicken body weight. While genome size (Fig. 4A) did not differ significantly between these two groups, average MCI of positively associated genome-reduced bacteria were slightly larger than those with negative association. Metatranscriptomics also revealed contrasting metabolic behaviour between both sets of bacteria (Fig. 4C). PCoA ordination and heatmap of expression profiles showed that the activity of UBA1242 was largely limited to the production of SCFAs (B04) and vitamin B1 (B0701). In contrast, UBA660 also expressed genes for the production of the essential amino acid lysine (B0211) and indole-3-acetate (B0805), and arginine degradation (D0509) (Fig. 4C).

## Discussion

The overall taxonomic composition and dynamics of the caecal microbiome observed in our study reflected the typical development described in broiler chickens reared in ventilated, climate controlled barns [8, 28]. However, the detailed analysis of microbial genome properties provided unique deeper insights into their functional characteristics, shedding light on the underlying processes driving the observed taxonomic composition and temporal dynamics.

### Functional profiling of the microbiome reveals a complex relationship with the taxonomic profile

The distillation of functional annotations into metabolic traits revealed a pronounced gradient of metabolic capacities among bacteria, which correlated with their genome sizes. The studied bacteria ranged from resource generalists—with large genomes, high metabolic capacities and a broad functional repertoire—to specialists, with reduced genomes and low metabolic capacities [29]. Most generalists with high metabolic capacities are well-known bacteria, such as *Blautia*, *Faecalibacterium* and *Bacteroides*, belonging to Lachnospirales, Oscillospirales, and Bacteroidales. As generalists, they can utilize multiple carbon and nitrogen sources, making them easier to cultivate and study [58, 59].

In contrast, the majority of clades grouping the specialist bacteria with low metabolic capacities are newly described taxa like UBA1242, RF32 and RF39 [60, 61]. These taxa comprise uncultured bacteria only recently discovered through metagenomics, although they seem to be widespread across animal gut environments [25, 60, 62–65], including humans [66, 67]. These largely unknown bacteria are not part of a distinct evolutionary branch like candidate phyla radiation (CPR) bacteria or Patescibacteria [68], but belong to several traditional taxonomic groups (e.g. Bacilli, Clostridia) or are closely related to them [60, 69]. However, they have undergone a severe genome reduction [70], making their functional capacities markedly different from those of their phylogenetic neighbors, with a significant number of auxotrophies [60, 71]. Some of these bacteria with reduced metabolic capacities are even incapable of performing fundamental biological functions, such as the biosynthesis of their own nucleic acids, which suggests that they largely rely on other bacteria or the host to obtain essential compounds [61]. An extreme example in our study can be found among Pseudomonadota, which include *Escherichia—*with over 5000 protein-coding genes—as well as the phylogenetically related lineage RF32, with less than 2000 genes.

### Microbiomes shift from generalists to specialists throughout chicken development

The temporal changes in community composition at phylum and order levels matched previously seen community development patterns in broilers reared in intensive systems. The initial dominance of Bacillota_A decreased progressively, while members of other phyla, led by Bacteroidota, grew their presence, leading to increasing alpha diversity metrics through time [8, 15]. However, we found that the metabolic features of the microbiota exhibited contrasting patterns compared to the community diversity metrics. While functional diversity increased through time, mirroring previously described patterns [17, 18], the average metabolic capacity of the caecal microbiome decreased. This phenomenon was explained by the functional characteristics of the bacteria that lost and gained representation between Days 7 and 35. Initially, the community was dominated by generalist bacteria, with high metabolic capacities yet functionally similar to each other, transitioning into a more diverse microbiota with a larger representation of specialist bacteria with low metabolic capacities but higher functional heterogeneity.

This transition from a community dominated by generalists to one with a higher share of specialists matches with a typical primary succession pattern [72]. Generalists with high metabolic flexibility can exploit a broader niche space, allowing them to survive under unstable and heterogeneous environmental conditions [29, 73, 74]. Chances for survival outside the host are therefore increased, likely contributing to their capacity to colonize the chicken gut soon after the eggs hatch [8]. Moreover, the ability to exploit a broader niche space also entails a higher metabolic independence. Without reliance on metabolic by-products produced by other microorganisms [75, 76], generalists dominate the initial stages of caecal microbiome development.

But generalism*—*which is achieved by retaining more genes in larger genomes*—*comes at a cost, as replicating larger genomes demands more energy for reproduction [77], and maintaining broader metabolic repertoires may limit the ability to optimize specific metabolic pathways [22]. In consequence, once the conditions are formed, specialist species tend to have competitive advantage over generalists and outcompete them in later successional stages [22]. In our study, as the microbiome matured, microorganisms with lower metabolic capacities, such as RF39, RF32, UBA1242, and TANB77, began to gain representation at the expense of bacteria with larger metabolic repertoires, such as Lachnospiraceae.

Such a transition is likely favored by the low dietary heterogeneity. In our experiment, although the feed formulation varied across three phases (starter, grower, and finisher), it remained relatively homogeneous [12] compared to the diverse diet of a free-ranging animal. Such stable conditions, with no disturbances, are especially favorable for specialist bacteria [78]. Additionally, commercial chicken diets are designed to facilitate energy absorption in the small intestine [79], leaving a reduced energy residue available for caecal bacteria. These conditions are also likely to favor low-capacity bacteria, due to their lower energy requirements compared to high-capacity bacteria [80].

### Genome-reduced bacteria might participate in the host's energy balance

Alpha diversity was negatively associated with chicken body weight at Day 35, as animals with higher diversities exhibited lower weights. This relationship has been previously reported [19], as has the association between low microbial diversity and obesity [81]. However, results are varied and contradictory, with very few taxa consistently associated with performance, likely due to many variables that are often not measured such as animal feed intake [21]. Despite the limitations of this study in establishing causality, we focused on the functional traits of the bacteria significantly associated with body weight to gain further insights into the potential host-microbiota interactions.

Most of the bacteria that exhibited a negative relationship with chicken body weight were generalists with large genomes and high capacity to synthesize and degrade a broad range of metabolic compounds. Although generalist bacteria can contribute to essential metabolic processes of hosts [82, 83], their beneficial effect might not be particularly relevant in the context of our study system for two reasons. First, broilers have undergone strong artificial selection to maximize the capacity to rapidly absorb and metabolize nutrients into skeletal tissue [84]. Second, feed formulations have been optimized to enable rapid and efficient absorption of nutrients by chickens [79]. In consequence, while a caecal microbiota with high metabolic capacity and activity to degrade complex compounds into more digestible metabolites is likely beneficial for chickens in general, in an intensive production context it might rather capture higher amounts of dietary energy, causing a deteriorating effect on performance traits. Contrarily, the promotion of genome-reduced bacteria whose metabolic activity is limited to a few key beneficial functions could provide similar functional characteristics with less energy consumption.

In fact only 10 bacteria were positively associated with chicken body weight, all specialists with reduced genomes belonging to the UBA660 clade. Research on RF39 order is still at early stages. The most distinctive feature is that they lack genes involved in de novo synthesis of nucleic acids, which have raised speculations about their parasitic or endosymbiotic relationship with vertebrates [61]. However, they are expected to have a thin wall, with the capacity of sporulating depending on the specific bacterium; unlike closely related RF20 and ML615J-28 orders, which exhibit a greater tendency to lose these features [71]. These observations could support a long and complex host-associated mutualistic symbiosis hypothesis, whereby bacteria would receive nucleic acids and other essential molecular compounds from the host in return to metabolic benefits [61]. However, the late-stage blooming of these bacteria during microbiome development also suggests they might rely on other bacteria for essential metabolites.

Gene expression analyses revealed that these bacteria, despite their reduced metabolic capacities, heavily expressed genes involved in SCFA production (i.e. acetate, butyrate, and propionate) in our experiments, supporting previous observations [60, 69]. Acetate is a common bacterial by-product linked to beneficial outcomes for animal biology in general [85, 86], and for chickens in particular [87]. Ecological theory suggests that the proportional influence of these bacteria in providing hosts with beneficial metabolites might be maximized compared to the high-capacity bacteria that dominate the early developmental stages, as specialists with traits better matched to their local environment, are expected to contribute disproportionately more to ecosystem function [22].

Nevertheless, not all genome-reduced bacteria were positively associated with chicken body weight. We also found 11 bacteria from the UBA1242 family (Bacillota_A) among the bacteria negatively associated with body weight. In fact, the presence of mid- to low-capacity bacteria in the negatively associated group probably precluded a significant association between chicken body weight and average metabolic capacity of the microbiome, suggesting a more complex relationship influenced by factors beyond genome size. The genome reduced taxa that associated positively and negatively with body weight were clearly distinguishable by their phylogenetic position: the negative group mostly belonged to UBA1242, whereas the positive group belonged exclusively to UBA660. While UBA1242 has received significantly less attention than UBA660 in the literature [61], our analyses revealed that UBA660 expressed more functions than UBA1242. Among the active pathways in UBA660 bacteria, those participating in the biosynthesis of lysine and methionine are of especial interest, since amino acids are usually one of the limiting components of animal growth in the feeds, and the most expensive nutrients to be added [79]. Although an excessive amount of amino acid in the diet might have adverse effects, a potential contribution from the gut microbiota could be beneficial [79]. In terms of indole-3-lactate (ILA) production, it could have a significant impact on host immunity and intestinal function, as some probiotics regulate intestinal homeostasis through indole derivative-induced AhR activation [88]. Future more exhaustive examination of the genome-scale metabolic networks of these specialist bacteria might provide further functional differences between the two groups.

## Conclusion

Multi-omic techniques enabled us to study species- and community-level functional characteristics and early-life temporal dynamics of the chicken caecal microbiome within an intensive production context. The diverse range of metabolic profiles within each phylum, the temporal dynamics of each bacterium, and the mismatch between community diversity and metabolic capacity, illustrates the need for further exploration of the ecological and functional aspects of gut microbiomes of farm animals. Moreover, the likely relevance of uncultured taxa (e.g. RF39, UBA1242) for microbiome development and animal growth surfaced, along with their pervasiveness across animal gut environments, render them worthy of deeper studies to unravel their parallel evolutionary processes of genome shrinkage in light of interactions with hosts and other microorganisms. Therefore, while this study provides significant insights into functional characteristics of the chicken caecal microbiome, it also opens avenues for further research to obtain commercially relevant bacterial biomarkers and/or new microbe-based solutions with the objective of achieving more precise husbandry approaches.

## Supplementary Material

sup_methods_figures_ycag091

new_supplementary_tables_ycag091

## Data accessibility

All raw DNA and RNA sequences, and the MAG catalogue are available under HoloFood’s umbrella project on ENA (Project ID: PRJEB43192). The MAG catalogue is available on PRJEB55374, raw metagenomic reads on PRJEB41323, PRJEB46550, PRJEB47613 and PRJEB39110, and raw metatranscriptomic reads on PRJEB52139. Data is also displayed in the HoloFood Data Portal [89]: www.holofooddata.org. Details related to sample processing statistics can be found in MGnify [90]: https://www.ebi.ac.uk/metagenomics/search/studies?query=holofood. Bioinformatic resources, including detailed scripts, data matrices and files have been archived in the following GitHub repository: https://github.com/alberdilab/chicken_genome_reduced_bacteria, with a snapshot stored in Zenodo with doi: https://doi.org/10.5281/zenodo.18457570. An html-rendered version of the repository is available at https://alberdilab.github.io/chicken_genome_reduced_bacteria.
