# Supplementary material for: Functional gut microbiota dynamics of generalist and specialist bacteria in association with chicken growth
Source: ISME Commun. 2026 Apr 10;6(1):ycag091. doi: 10.1093/ismeco/ycag091 (PMC13155120; doi:10.1093/ismeco/ycag091)

Supplementary methods and figures

Laboratory sample distribution and the strategy for adjusting molarity.

Laboratory processing was conducted in batches of 90 samples, along with 6 negative controls, including two extraction, two library preparation and two library indexing blanks. Samples within each batch were randomised using a custom script.

When the required number of cycles exceeded 12 cycles, the library preparation was repeated for considering that library-preparation inefficiency could yield technically biased results. Libraries were subsequently indexed using unique dual tags and the required number of PCR cycles, and bead-purified before the final quality-check using a Fragment Analyser (Agilent) with NGS fragment assay. Libraries with expected fragment-size distributions and molarities were equimolarly pooled for sequencing. Libraries with too low molarities were re-indexed and pooled to achieve the desired molarity. Libraries exhibiting unusual fragment distributions and large adaptor dimers were re-built to ensure maximum quality of generated data.

Generation of the MAG catalogue

Data from 261 chicken gut metagenomic samples sequenced with MGISeq-2000 were used to generate the caecal MAG catalogue. *De novo* metagenomic assemblies were generated using the MGnify assembly pipeline [1]. The assembly tool MetaSPAdes [2] was used preferentially for single-run assemblies, with MEGAHIT [3] being used for co-assemblies where the memory requirements for MetaSPAdes were too high. Groups of samples prioritised for co-assembly were selected by hierarchical clustering based on Jaccard distance between low-quality bins generated by single assembly. Contigs shorter than 500 base pairs were excluded, and further host, human and PhiX decontamination was performed post-assembly with blastn [4]. Contig binning was performed with metaWRAP's binning and bin_refinement modules. Genome quality was assessed with checkM [5] to retain those genomes with completeness >50%, contamination <5%, and QS >50 (where QS = completeness - 5*contamination). Genomes were de-replicated using an Average Nucleotide Identity (ANI) of 95%, and 30% alignment fraction to generate species-level clusters using dRep [6]. GUNC [7] was used to identify potentially chimeric genomes for removal, with the parameters clade separation score >0.45, contamination >0.05, and reference representation score >0.5. Taxonomic assignment was performed with GTDB-tk (release 207) [8]. A dedicated publication that will address the procedures employed for

generating the MAG catalogue used in this study is under preparation, and the employed code is available at Workflowhub (https://workflowhub.eu/programmes/28).

Details about the statistical approach for assessing the significance of response variables in alpha and beta diversities.

LMMs were fitted through the R package nlme *[9]*, using the components of alpha diversity as response variables and trial (categorical variable with two levels), chicken age (numeric variable), sex (categorical variable with two levels), genetic line (categorical variable with two levels) and treatment (categorical variable with three levels) as fixed explanatory variables. A pen-level random intercept was included in the models to specify that chicken individuals were nested within pens. Log-transformed sequencing depth was also included as explanatory variable in all alpha diversity models to account for the varying sequencing effort across samples. PERMANOVAs were fitted through R package vegan [10]. Neutral, phylogenetic and functional dissimilarity matrices were included as responses in the PERMANOVAs and trial, chicken age, sex, genetic line and treatment were included as explanatory variables. Permutations were constrained to within pens to account for the nested structure of the data. Very weak effects of the categorical variables sex, genetic line and treatment were observed in the PERMANOVA and LMM analyses, and none of these variables showed significant interactions with chicken age. Therefore, they were included in the rest of the analyses of this study to account for their possible confounding effect, but their effect was not interpreted.

Details for hierarchical modelling of species communities (HMSC) framework.

Raw counts of bacterial MAGs (weighted by the size of their genomes) were used as response variables in the model and trial, chicken age, sex, genetic line and treatment were included as fixed explanatory variables. Additionally, log-transformed library size was included as an extra explanatory variable to account for the compositionality of the data. To account for the nested study design a pen-level random effect was included in the model. The response variables were scaled to mean zero and unit variance and the log-normal model was applied. Variance partitioning showed that most of the microbiota variance occurred between time points (Supplementary Table S7). To assess whether the MAGs increased, decreased or remained stable over time a posterior support of 0.95 was used as a significance threshold: If 95% of the posterior distribution of the slope parameter linking the MAG abundance and chicken age was positive the MAG was considered to increase significantly, whereas if it was negative the MAG was considered to decrease significantly.

We fitted the models assuming the default priors and sampled the posterior distribution running four Markov Chain Monte Carlo (MCMC) chains, each of which was run for 3,750 iterations, of which 1,250 were discarded as burn-in. We thinned by 10 to obtain a total of 250 posterior samples per chain and 1000 posterior samples in total. We ensured MCMC convergence by measuring the potential scale reduction factor [11] for the beta parameters (measuring the response of the MAGs to the fixed effects). To examine whether the responses of the MAGs showed a phylogenetic signal to time, we included in the analysis a phylogenetic correlation matrix C among the MAGs computed from the above-mentioned phylogenetic tree. In HMSC, the phylogenetic signal is measured using the parameter ρ, which takes values from 0 to 1, a value of 0 meaning no phylogenetic signal in the response to fixed effects, and a value of 1 meaning a completely phylogenetically structured response. Then, to measure the phylogenetic scale at which the response of the bacterial MAGs to chicken age was structured, we built a phylogenetic correlogram linking the MAGs' associations with time and the phylogenetic distance between MAGs using the R package phylosignal [12]. Finally, to compute the predictive power of the model we calculated the R^2 using two-fold cross-validation in two alternative ways. In the first case, $CV_{standard}$ hereafter, the samples were divided into the training and testing set randomly, hence the samples from both replicate trials A and B were used to train the model when making the predictions. In the second case, CV_{trial} hereafter, the samples from experimental trials A and B were assigned separately to the training and testing sets, thus to make the predictions for trial A the model was only trained with samples from trial B, and, vice versa, to make the predictions for trial B the model was only trained with samples from trial A. Comparing R^2 from $CV_{standard}$ with R^2 from CV_{trial} allowed us evaluating whether the trends observed from our experimental trials were similar and thus generalizable between them. Both CV types yielded very similar R^2 values ($CV_{standard}$ = 0.54; CV_{trial} = 0.51) indicating that our results were consistent and generalizable between the two replicate trials.

Bibliography

1. Richardson L, Allen B, Baldi G, Beracochea M, Bileschi ML, Burdett T, et al. MGnify: the microbiome sequence data analysis resource in 2023. *Nucleic Acids Res* 2023; **51**: D753–D759.
2. Nurk S, Meleshko D, Korobeynikov A. metaSPAdes: a new versatile metagenomic assembler. *Genome* 2017.
3. Li D, Liu C-M, Luo R, Sadakane K, Lam T-W. MEGAHIT: an ultra-fast single-node solution for large and complex metagenomics assembly via succinct de Bruijn graph. *Bioinformatics* 2015; **31**: 1674–1676.
4. Chen Y, Ye W, Zhang Y, Xu Y. High speed BLASTN: an accelerated MegaBLAST search tool. *Nucleic Acids Res* 2015; **43**: 7762–7768.
5. Parks DH, Imelfort M, Skennerton CT, Hugenholtz P, Tyson GW. CheckM: assessing the quality of microbial genomes recovered from isolates, single cells, and metagenomes. *Genome Res* 2015; **25**: 1043–1055.
6. Olm MR, Brown CT, Brooks B, Banfield JF. dRep: a tool for fast and accurate genomic comparisons that enables improved genome recovery from metagenomes through de-replication. *ISME J* 2017; **11**: 2864–2868.
7. Orakov A, Fullam A, Coelho LP, Khedkar S, Szklarczyk D, Mende DR, et al. GUNC: detection of chimerism and contamination in prokaryotic genomes. *Genome Biol* 2021; **22**: 178.
8. Chaumeil P-A, Mussig AJ, Hugenholtz P, Parks DH. GTDB-Tk: a toolkit to classify genomes with the Genome Taxonomy Database. *Bioinformatics* 2019; **36**: 1925–1927.
9. Pinheiro J, Bates D, DebRoy S, Sarkar D. R Core Team (2021). nlme: Linear and Nonlinear Mixed Effects Models. R package version 3.1-152. 2021.
10. Oksanen J, Blanchet FG, Friendly M, Kindt R, Legendre P, McGlinn D, et al. vegan: Community Ecology Package. R package version 2.5--7. 2020. 2022.
11. Tikhonov G, Opedal ØH, Abrego N, Lehikoinen A, de Jonge MMJ, Oksanen J, et al. Joint species distribution modelling with the r-package Hmsc. *Methods Ecol Evol* 2020; **11**: 442–447.
12. Keck F, Rimet F, Bouchez A, Franc A. phylosignal: an R package to measure, test, and explore the phylogenetic signal. *Ecol Evol* 2016; **6**: 2774–2780.

Figure S1. Impact of genome completeness correction on the metabolic capacity index. a) The genome completeness correction (Eisenhofer et al. 2023) was developed to reduce the underestimation of functional capabilities derived from the incomplete recovery of bacterial genomes. As a result, the method increases the mean MCI of incomplete genomes and it is effective in reducing the technical correlation between completeness and mean MCI of MAGs. Spearman correlation decreased substantially after applying the completeness correction (ρ = 0.63 without correction vs. ρ = 0.22 with correction). However, the method increases the capabilities of functions that were present or absent indistinguishably. As a result, it over-inflates the capabilities to produce many metabolites that were absent in the corrected MAGs. This is particularly problematic for incomplete MAGs with small genomes (e.g. RF39 - yellow and UBA1242 - brown), which are the main focus of our study. For those specific cases, the MAG completeness becomes the main driver of mean MCI, introducing a severe bias to their functional profile (ρ = 0.55 without correction vs. ρ = -0.93 with correction. In light of these observations, we decided to avoid the completeness correction step. **b)** At the community-level, applying the correction did not alter the overall temporal trend, and MCI continued to decrease over time. The only difference was that values were slightly higher.

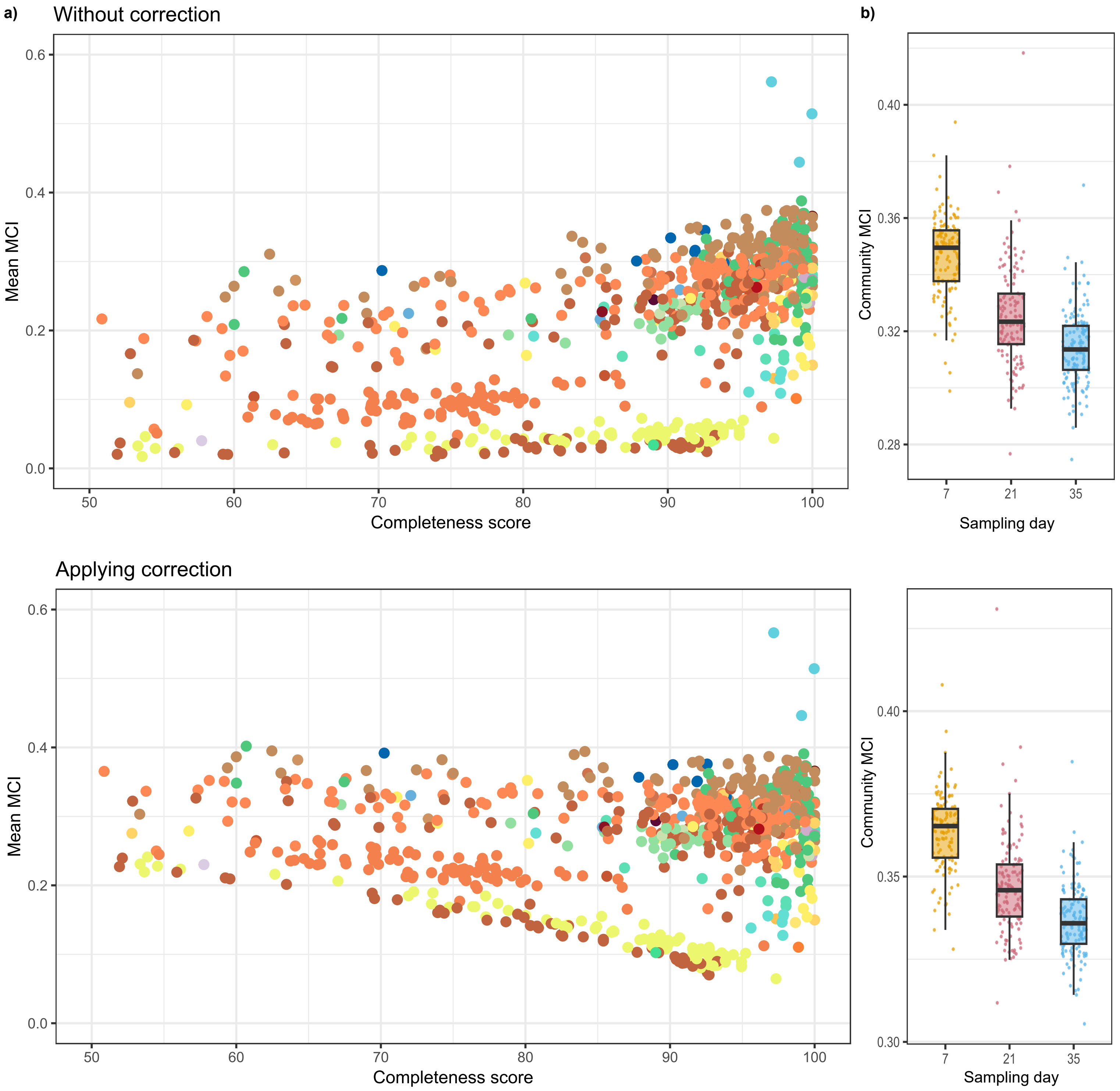

Figure S2. MAG average metabolic capacity index (MCI) and genome length (Mbp) by taxonomic phylum and order.

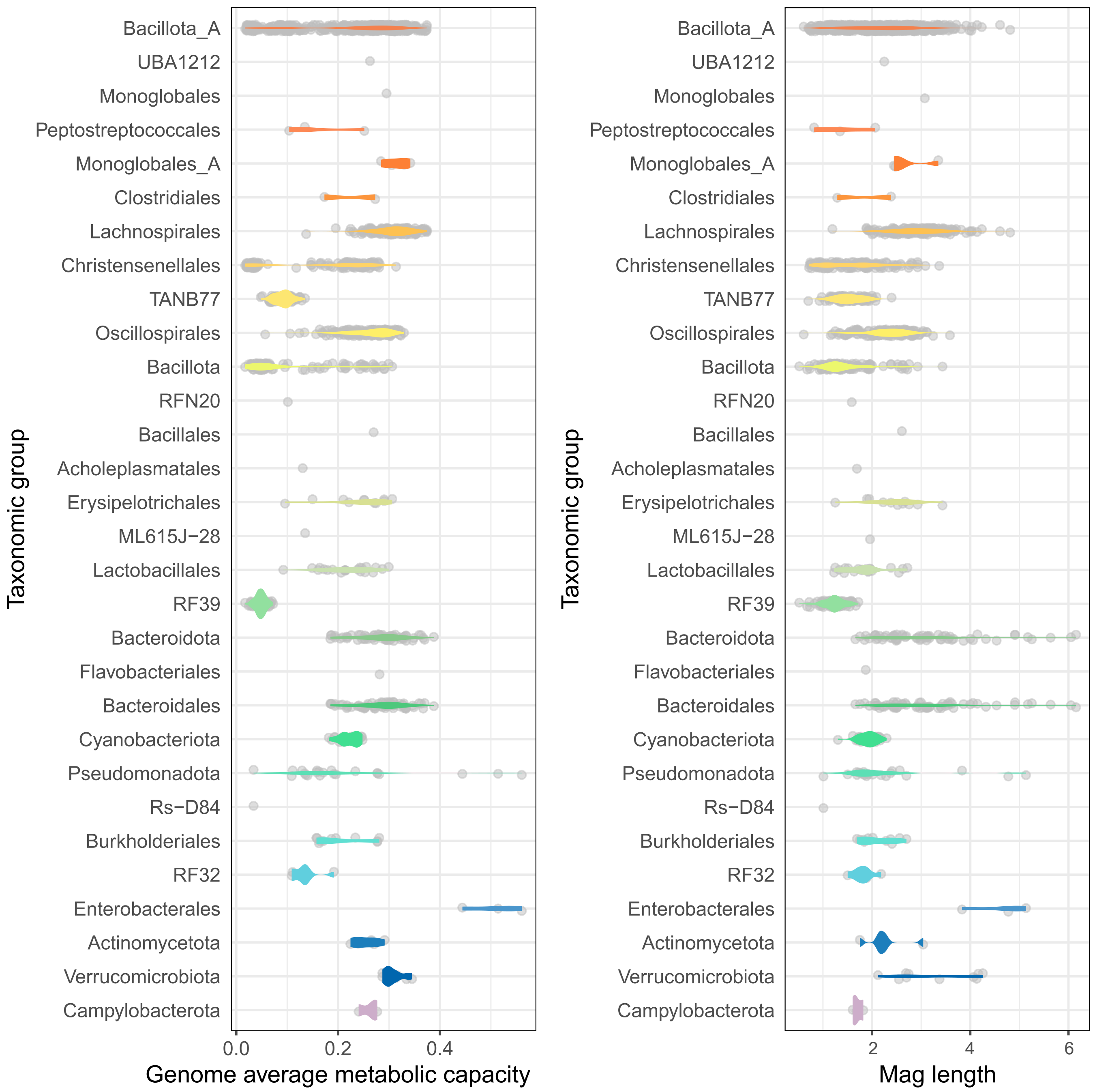

Figure S3. Temporal differences for neutral, phylogenetic and functional beta diversities comparing animals from the same trial and same pen.

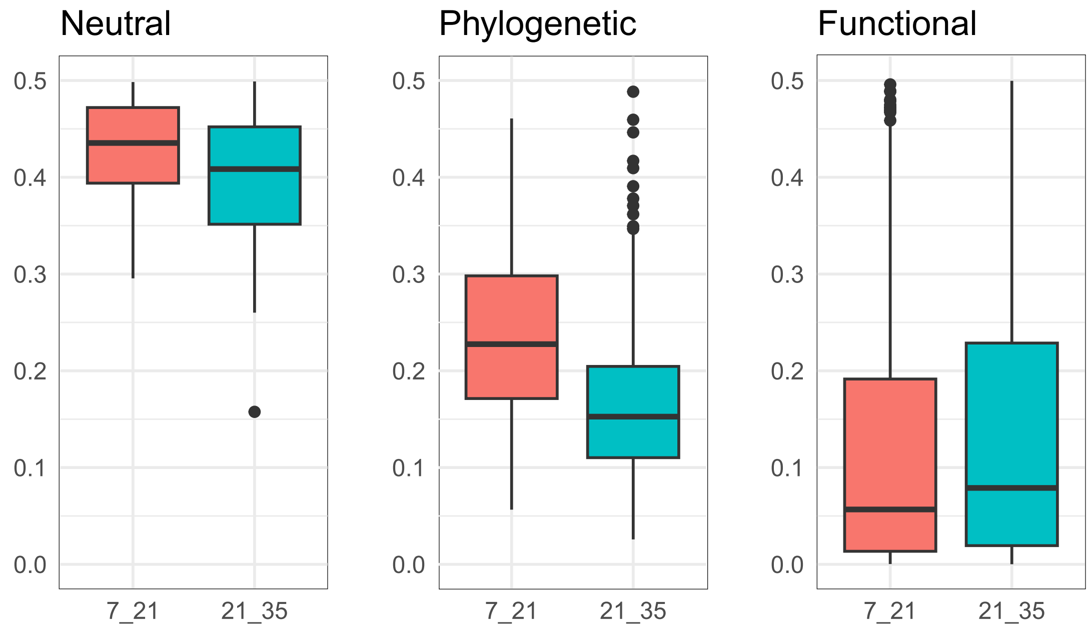

Figure S4. Temporal differences for neutral, phylogenetic and functional beta diversities comparing animals from the same trial (CA, CB) and sampling day.

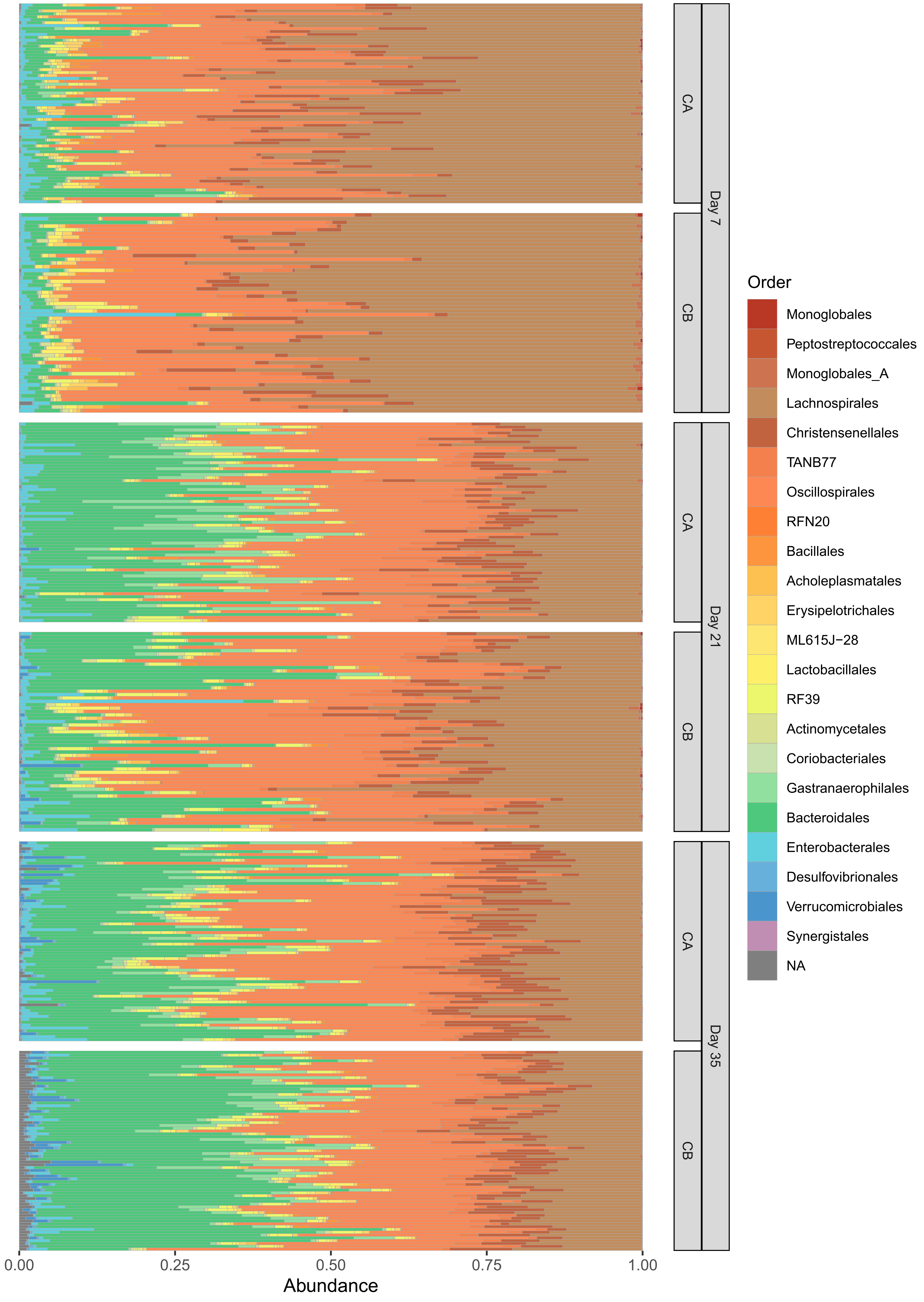

Figure S5. Comparative biosynthesis capability between bacteria negatively and positively associated with chicken body weight at day 35. Each graph refers to a function block: B01 - Nucleic acid biosynthesis, B02 - Amino acid biosynthesis, B03 - Amino acid derivative biosynthesis, B04 - SCFA biosynthesis, B06 - Organic anion biosynthesis, B07 - Vitamin biosynthesis, B08 - Aromatic compound biosynthesis, B09 - Metallo-phore biosynthesis, B10 - Antibiotic biosynthesis.

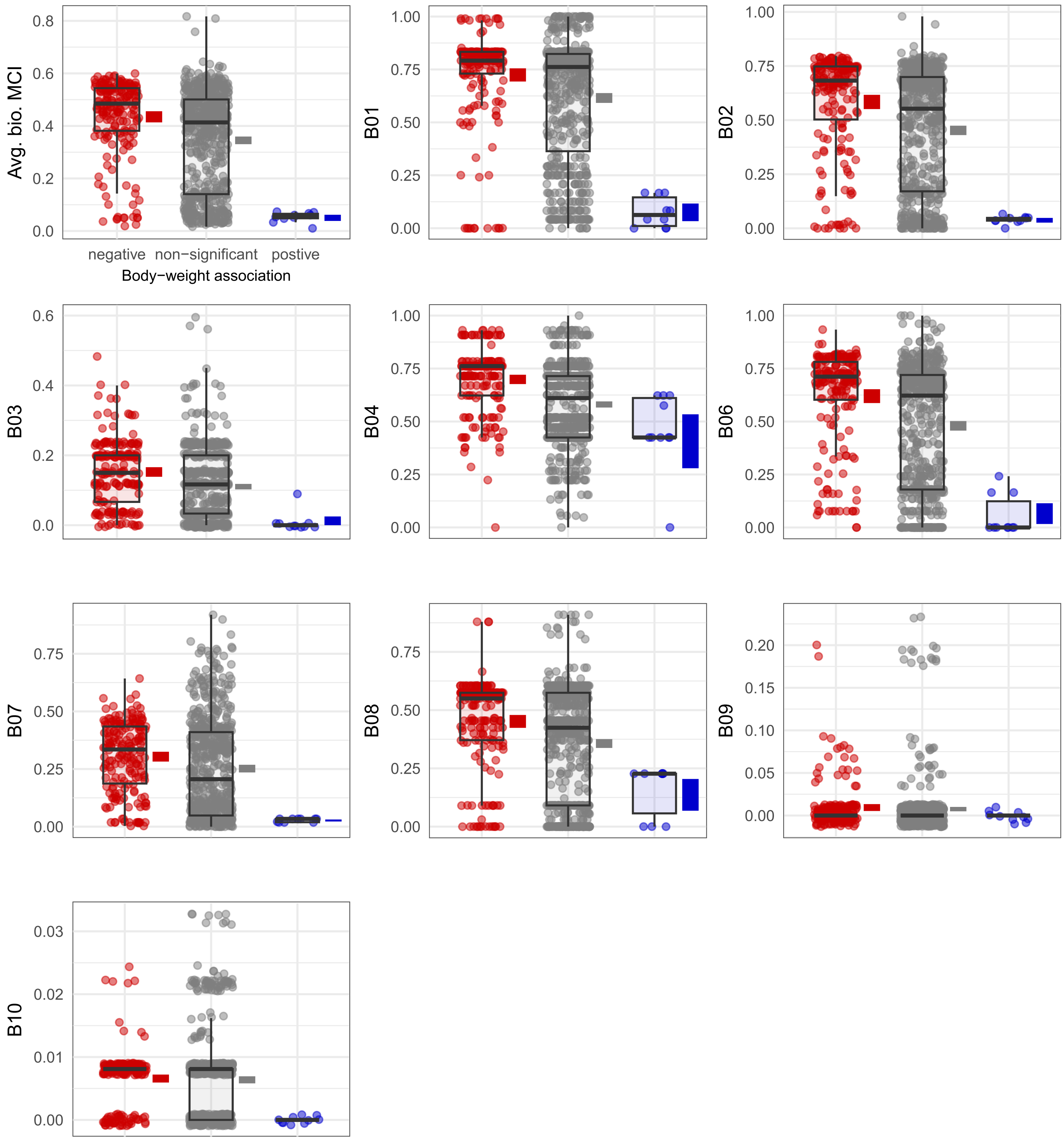

Figure S6. Comparative biosynthesis capability between bacteria negatively and positively associated with chicken body weight at day 35. Each graph refers to a function. D01 - Lipid degradation, D02 - Polysaccharide degradation, D03 - Sugar degradation, D04 - Protein degradation, D05 - Amino acid degradation, D06 - Nitrogen compound degradation, D07 - Alcohol degradation, D08 - Xenobiotic degradation, D09 - Antibiotic degradation.

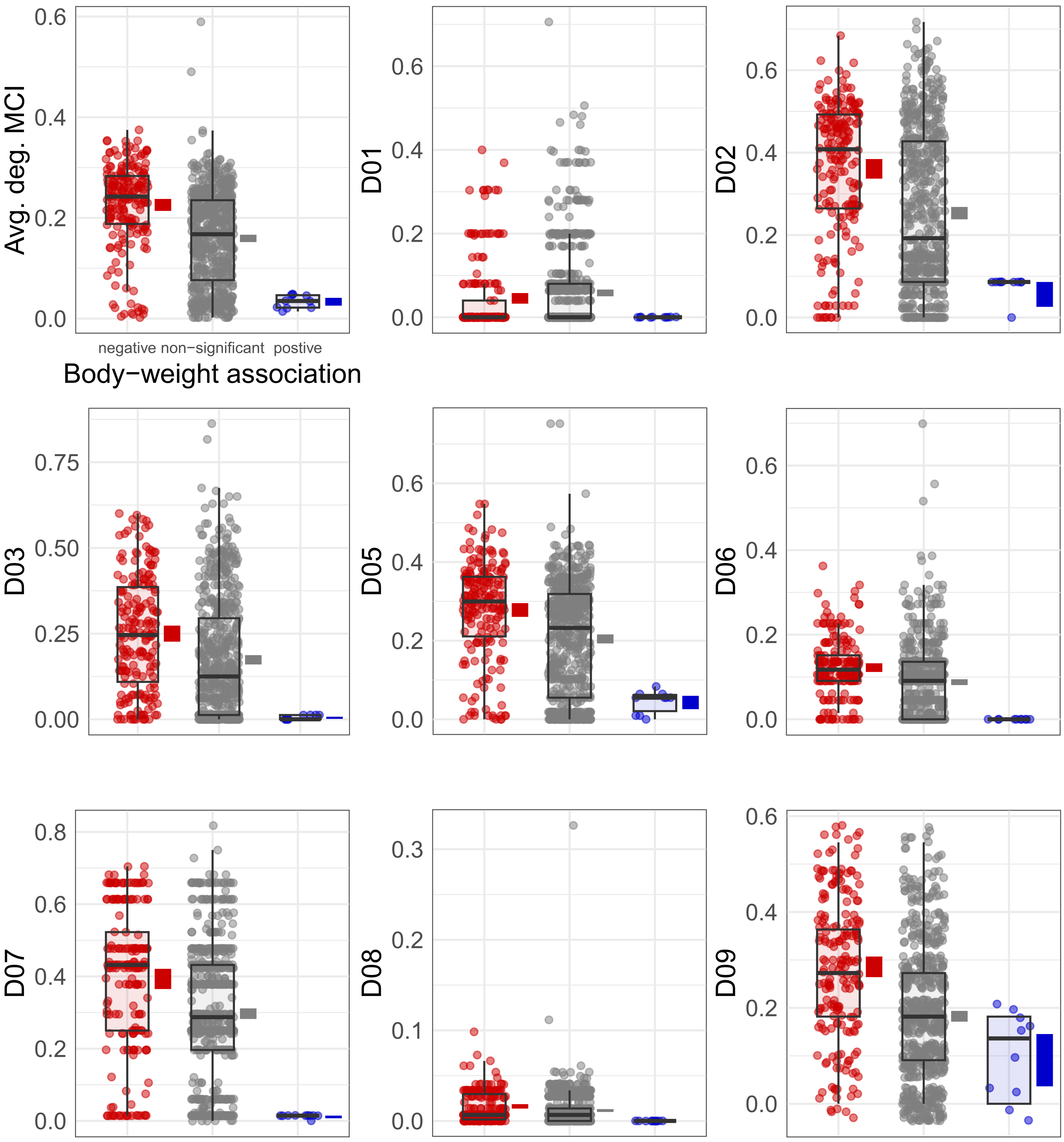

Figure S1. Impact of genome completeness correction on the metabolic capacity index. a) The genome completeness correction (Eisenhofer et al. 2023) was developed to reduce the underestimation of functional capabilities derived from the incomplete recovery of bacterial genomes. As a result, the method increases the mean MCI of incomplete genomes and it is effective in reducing the technical correlation between completeness and mean MCI of MAGs. Spearman correlation decreased substantially after applying the completeness correction (ρ = 0.63 without correction vs. ρ = 0.22 with correction). However, the method increases the capabilities of functions that were present or absent indistinguishably. As a result, it over-inflates the capabilities to produce many metabolites that were absent in the corrected MAGs. This is particularly problematic for incomplete MAGs with small genomes (e.g. RF39 - yellow and UBA1242 - brown), which are the main focus of our study. For those specific cases, the MAG completeness becomes the main driver of mean MCI, introducing a severe bias to their functional profile (ρ = 0.55 without correction vs. ρ = -0.93 with correction. In light of these observations, we decided to avoid the completeness correction step. **b)** At the community-level, applying the correction did not alter the overall temporal trend, and MCI continued to decrease over time. The only difference was that values were slightly higher.

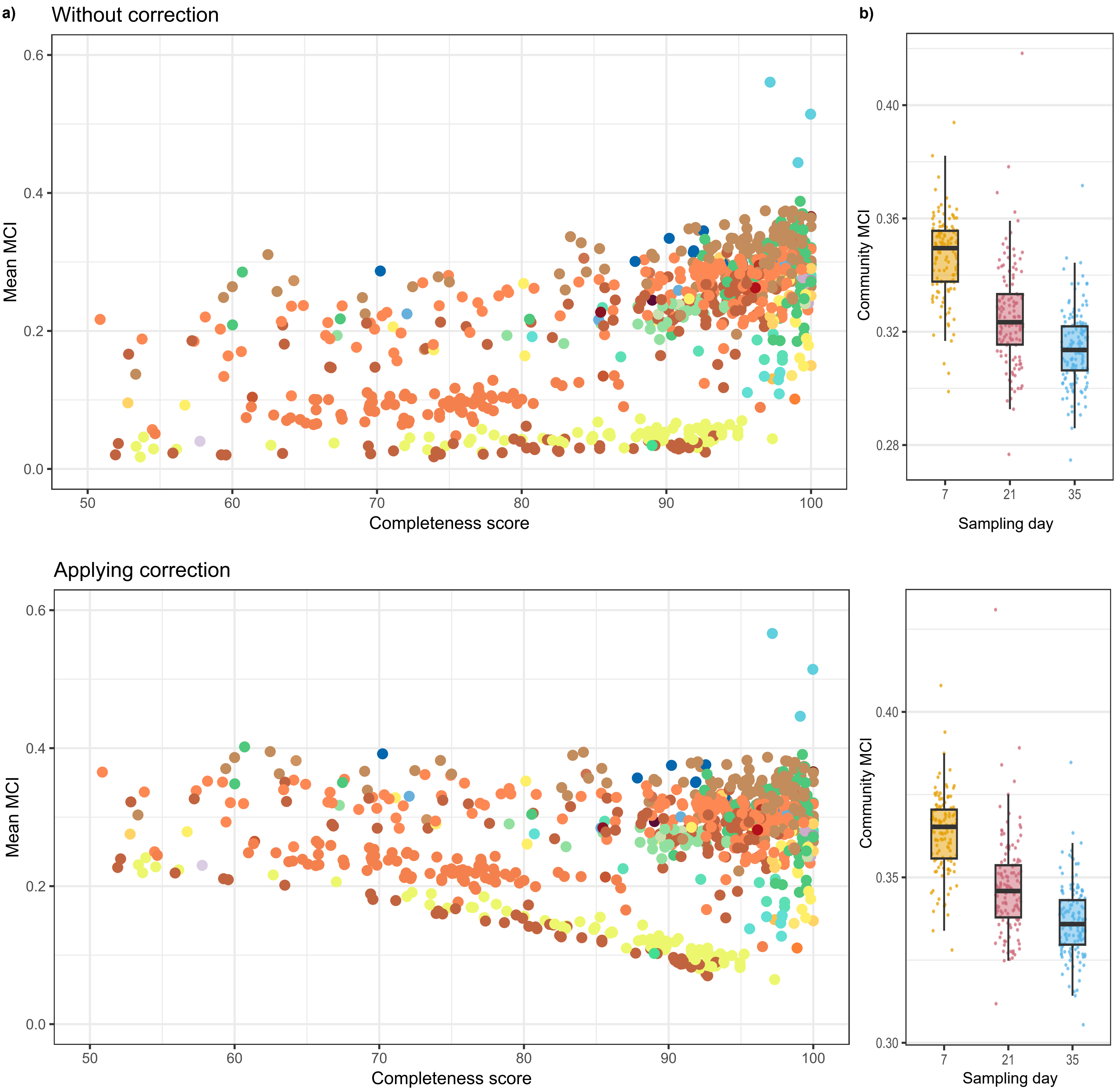

Figure S2. MAG average metabolic capacity index (MCI) and genome length (Mbp) by taxonomic phylum and order.

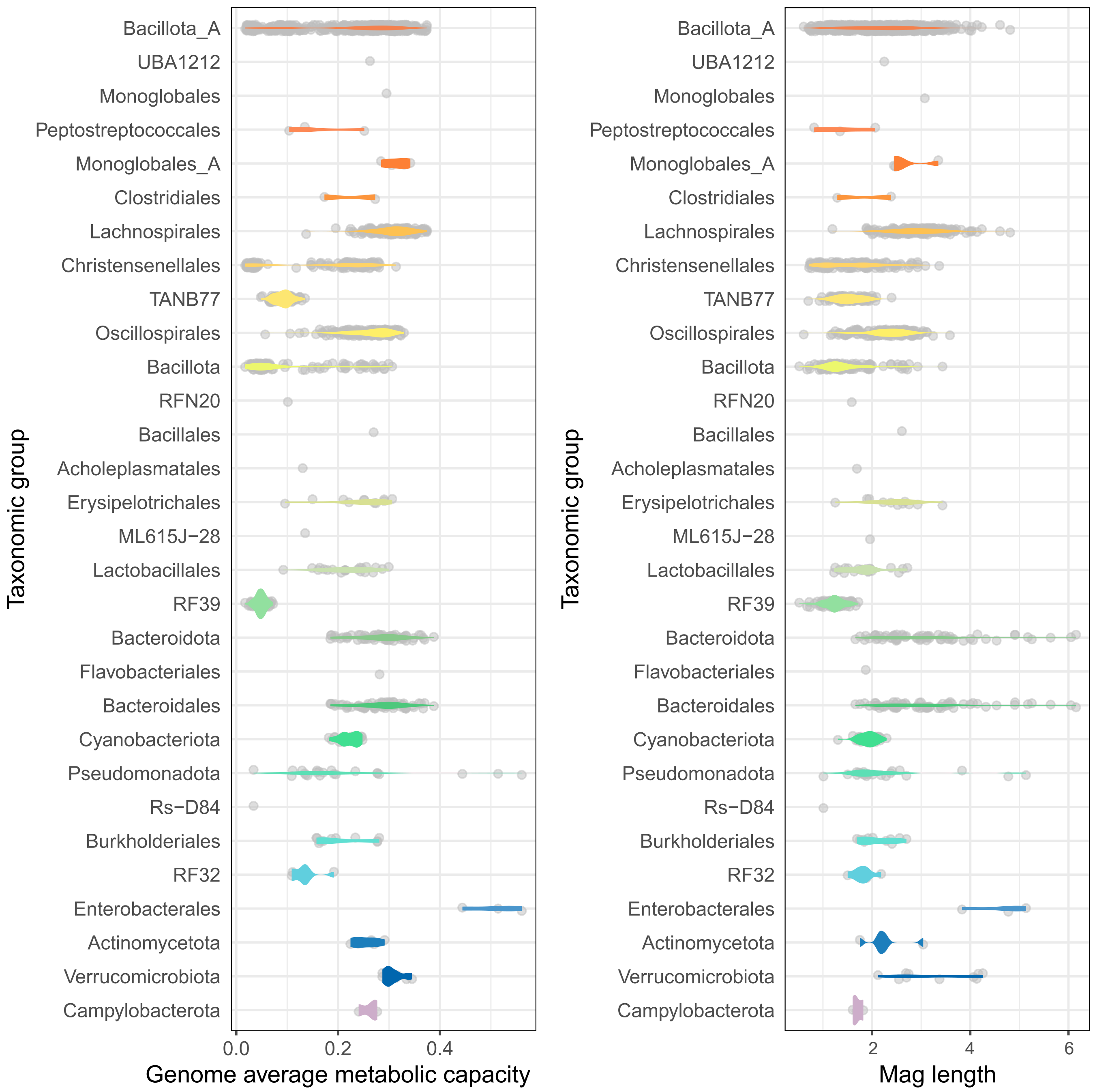

Figure S3. Temporal differences for neutral, phylogenetic and functional beta diversities comparing animals from the same trial and same pen.

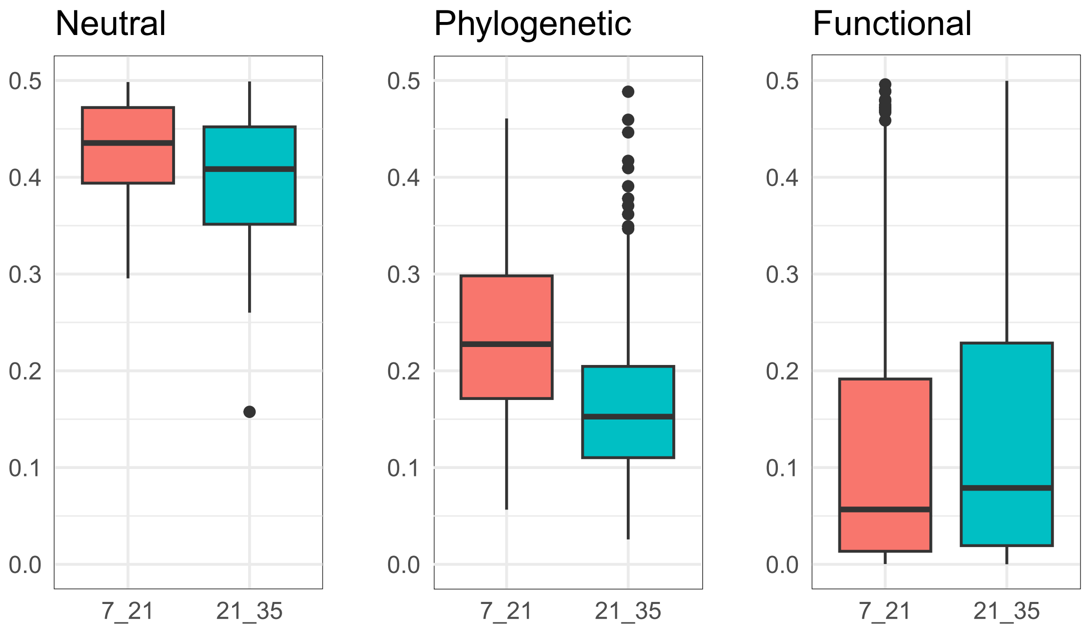

Figure S4. Temporal differences for neutral, phylogenetic and functional beta diversities comparing animals from the same trial (CA, CB) and sampling day.

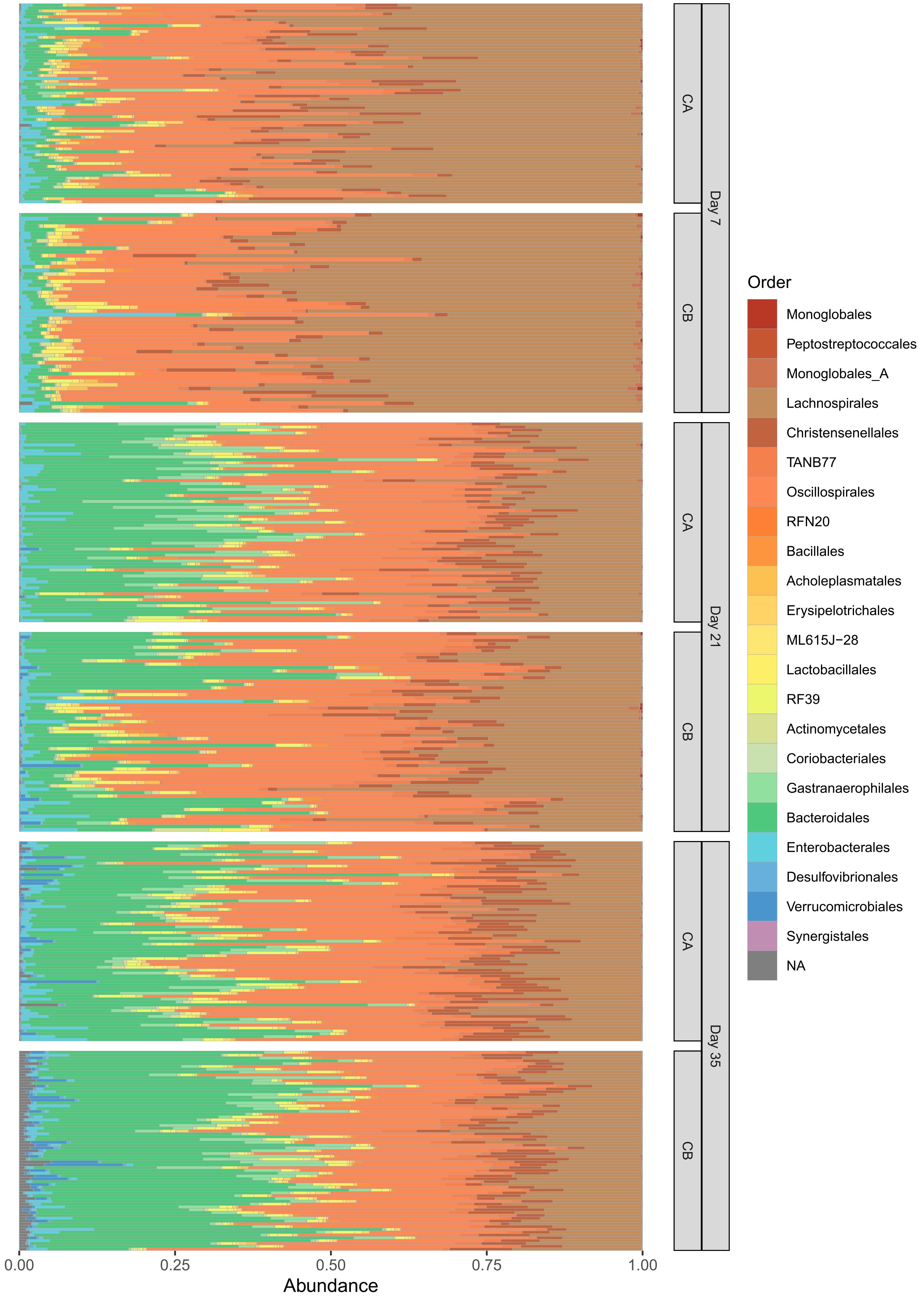

Figure S5. Comparative biosynthesis capability between bacteria negatively and positively associated with chicken body weight at day 35. Each graph refers to a function block: B01 - Nucleic acid biosynthesis, B02 - Amino acid biosynthesis, B03 - Amino acid derivative biosynthesis, B04 - SCFA biosynthesis, B06 - Organic anion biosynthesis, B07 - Vitamin biosynthesis, B08 - Aromatic compound biosynthesis, B09 - Metallo-phore biosynthesis, B10 - Antibiotic biosynthesis.

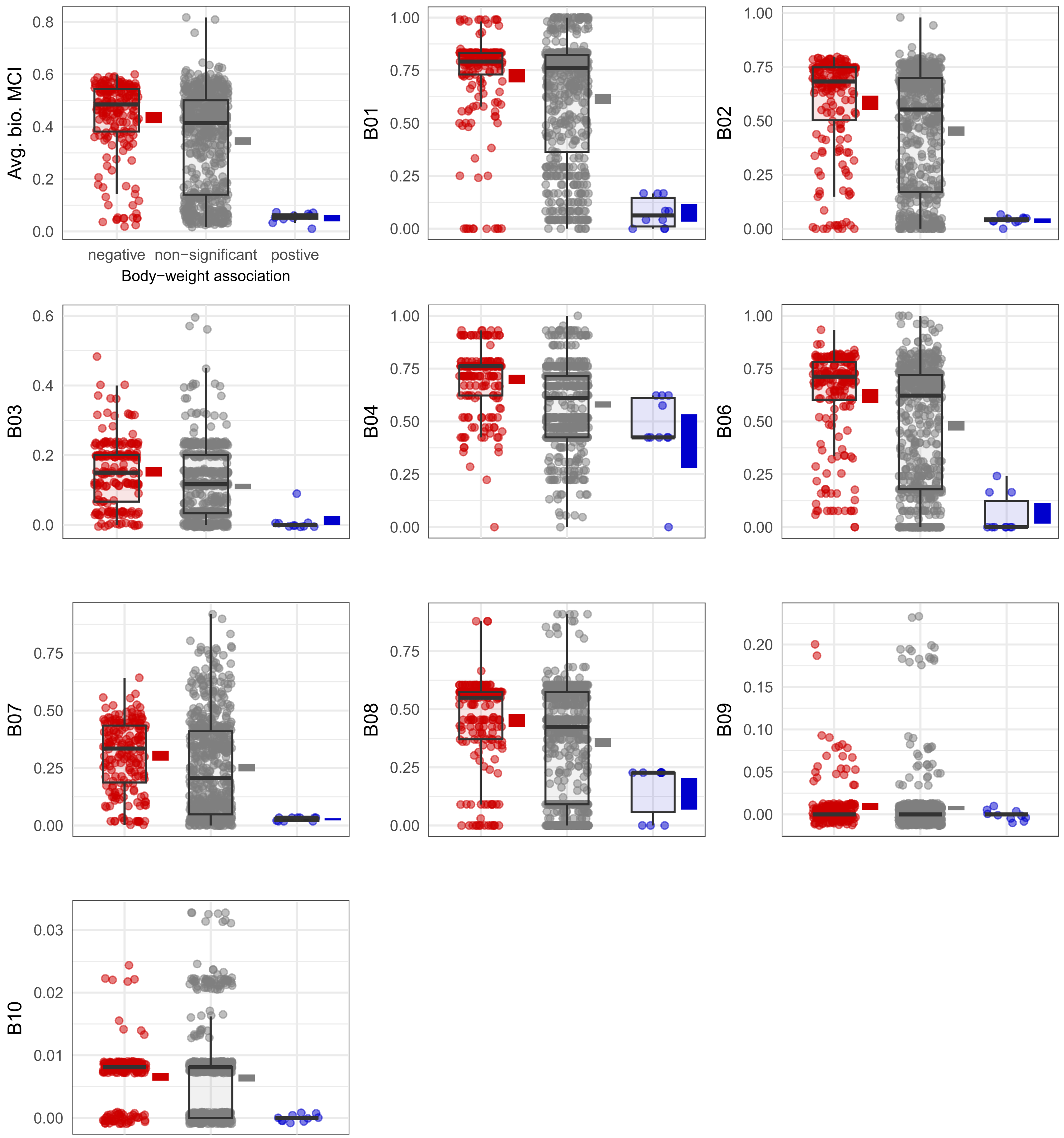

Figure S6. Comparative biosynthesis capability between bacteria negatively and positively associated with chicken body weight at day 35. Each graph refers to a function. D01 - Lipid degradation, D02 - Polysaccharide degradation, D03 - Sugar degradation, D04 - Protein degradation, D05 - Amino acid degradation, D06 - Nitrogen compound degradation, D07 - Alcohol degradation, D08 - Xenobiotic degradation, D09 - Antibiotic degradation.

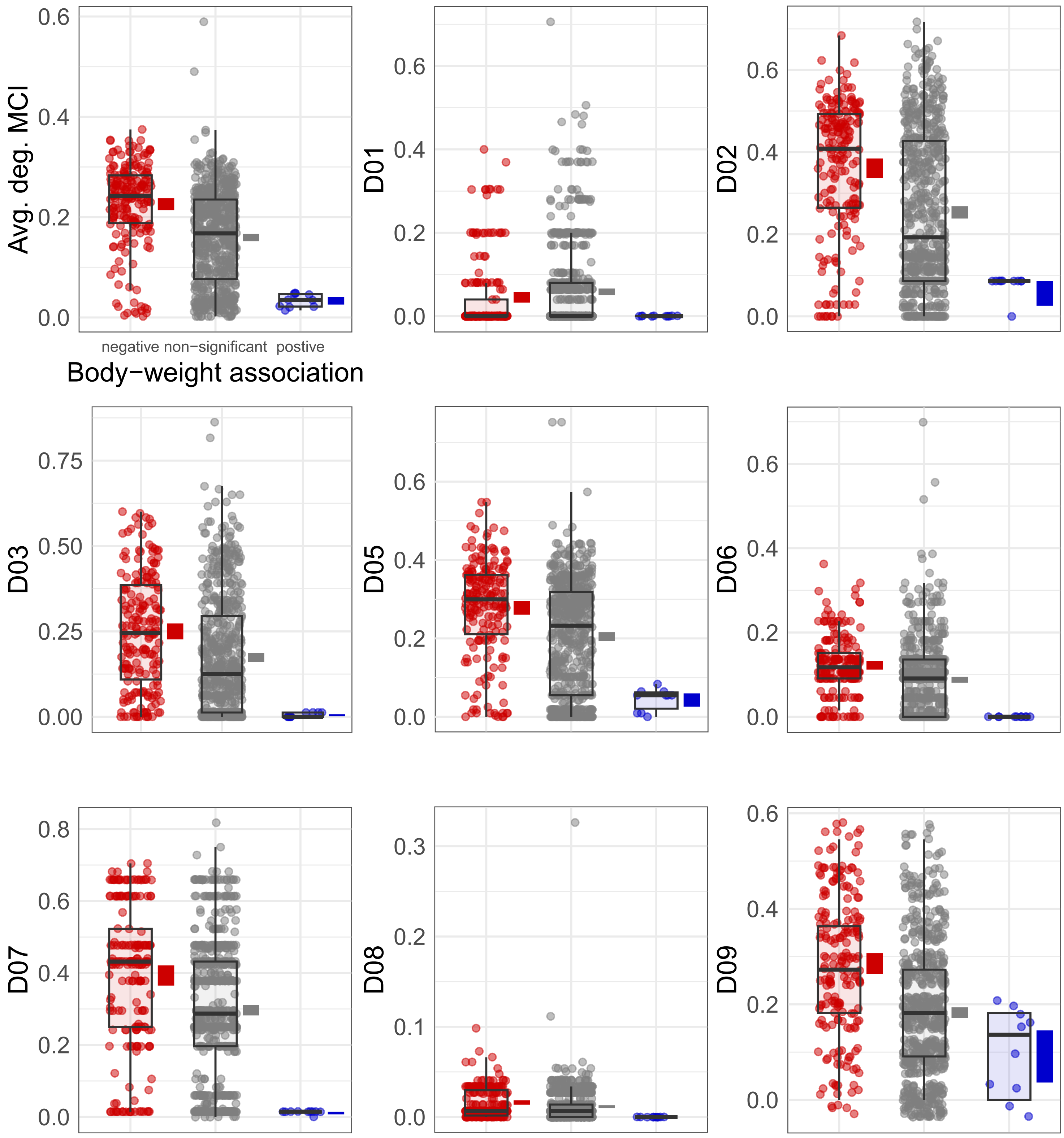

Supplement: sup_methods_figures_ycag091 [file sup_methods_figures_ycag091.pdf]
